# Draft Genome Sequences of Gammaproteobacterial Methanotrophs Isolated from Marine Ecosystems

**DOI:** 10.1128/genomeA.01629-15

**Published:** 2016-01-21

**Authors:** James D. Flynn, Hisako Hirayama, Yasuyoshi Sakai, Peter F. Dunfield, Martin G. Klotz, Claudia Knief, Huub J. M. Op den Camp, Mike S. M. Jetten, Valentina N. Khmelenina, Yuri A. Trotsenko, J. Colin Murrell, Jeremy D. Semrau, Mette M. Svenning, Lisa Y. Stein, Nikos Kyrpides, Nicole Shapiro, Tanja Woyke, Françoise Bringel, Stéphane Vuilleumier, Alan A. DiSpirito, Marina G. Kalyuzhnaya

**Affiliations:** a San Diego State University, Department of Biology, San Diego, California, USA; b Department of Subsurface Geobiological Analysis and Research, Japan Agency for Marine-Earth Science & Technology (JAMSTEC), Yokosuka, Kanagawa, Japan; c Division of Applied Life Sciences, Graduate School of Agriculture, Kyoto University, Kitashirakawa-Oiwake, Sakyo-ku, Japan; d Department of Biological Sciences, University of Calgary, Calgary, Alberta, Canada; e Division of Math & Natural Sciences, Queens College in the City University of New York, Flushing, New York, USA; f Institute of Crop Science and Resource Conservation—Molecular Biology of the Rhizosphere, University of Bonn, Bonn, Germany; g Department of Microbiology, IWWR, Faculty of Science, Radboud University, Nijmegen, The Netherlands; h GK Skryabin Institute of Biochemistry and Physiology of Microorganisms, Russian Academy of Sciences, Pushchino, Russian Federation; i School of Environmental Sciences, University of East Anglia, Norwich Research Park, Norwich, United Kingdom; j Department of Civil and Environmental Engineering, The University of Michigan, Ann Arbor, Michigan, USA; k Department of Arctic and Marine Biology, UiT, The Arctic University of Norway, Tromsø, Norway; l Department of Biological Sciences, University of Alberta, Edmonton, Alberta, Canada; m DOE Joint Genome Institute, Walnut Creek, California, USA; n Department of Microbiology, Genomics and the Environment, Université de Strasbourg, UMR 7156 CNRS, Strasbourg, France; o Roy J. Carver Department of Biochemistry, Biophysics and Molecular Biology, Ames, Iowa, USA

## Abstract

The genome sequences of *Methylobacter marinus* A45, *Methylobacter* sp. strain BBA5.1, and *Methylomarinum vadi* IT-4 were obtained. These aerobic methanotrophs are typical members of coastal and hydrothermal vent marine ecosystems.

## GENOME ANNOUNCEMENT

Microbial methane oxidation is one of the key drivers of oxygen consumption in marine sediments and the overlaying water column (1). Methanotrophic bacteria are the primary producers of many cold and hot seep ecosystems (2, 3). Here, we report three genome sequences of gammaproteobacterial methanotrophs isolated from three marine ecosystems. *Methylobacter marinus* A45 (a methanol-adapted strain, formerly *Methylomonas methanica* A4, ACM 4717) was isolated from sewage outfall sediment near Los Angeles, CA (4). *Methylobacter* sp. strain BBA5.1 was isolated from the surface layer of estuary sediment collected at low tide near Newport, Bay Estuary (CA) (5). *Methylomarinum vadi* IT-4 (= JCM 13665^T^ = DSM 18976^T^) was isolated from a microbial mat of a shallow submarine hydrothermal system near Taketomi Island, Okinawa, Japan (6).

DNA samples from the three strains were prepared using the standard phenol-chloroform method (7). DNA sequence data were obtained at the Joint Genome Institute using a combination of PacBio (8) and Illumina (9) technologies, and draft genome sequences were assembled. The computational tools used for genome sequencing and assembly are listed in Table 1.

**TABLE 1:** General genome statistics and accession numbers

Species	Sequencing platform(s)	Genome assembly and annotation	Genome coverage (×)	Genome size (Mb)	No. of scaffolds (no. of contigs)	Core (accessory) metabolic pathways^a^	NCBI accession no.
*M. marinus* A45	Illumina	Velvet 1.1.05, AllPaths, Phrap 4.24, Prodigal 2.5	1,237	4.99	9 (49)	pMMO, pXmo, Mxa, XoxF1, XoxF2, H_4_F, H_4_MPT, FDH, RuMP, EMP, EDD, dPPP, PPP, pSC, TCA	ARVS00000000
*Methylobacter* sp. BBA5.1	Illumina, PacBio RS	AllPaths, Prodigal 2.5	290	5.07	87 (91)	pMMO, pXmo, Mxa, XoxF1, XoxF2, H_4_F, H_4_MPT, FDH, RuMP, EMP, EDD, dPPP, PPP, pSC, TCA	JQKS00000000
*M. vadi* IT-4	Illumina, PacBio RS	Prodigal 2.5	272	4.33	1 (1)	pMMO, Mxa, XoxF, H_4_F, H_4_MPT, FDH, RuMP, EMP, EDD, dPPP, PPP, pSC, TCA	JPON00000000

^a^ dPPP, dissimilatory pentose-phosphate pathway; EDD, Entner-Doudoroff pathway; EMP, Embden-Meyerhof-Parnas pathway; FDH, formate dehydrogenases; H_4_F, folate-linked C_1_ transfer; H_4_MPT, methanopterin-linked C_1_ transfer; Mxa, PQQ-linked methanol dehydrogenases; pMMO, membrane-bound methane monooxygenase; pSC, partial serine cycle; pXmo, methane/ammonia monooxygenase-related proteins of unknown function; PPP, pentose-phosphate pathway; RuMP, assimilatory ribulose monophosphate pathway; Xox, PQQ-linked methanol and formaldehyde dehydrogenases (i.e., no evidence for the glyoxylate regeneration pathway was found); TCA, tricarboxylic acid cycle.

All three sequenced marine methanotrophs are obligate methane and methanol utilizers. All three genomes harbor genes typical for type I methanotrophs, including genes encoding particulate methane monooxygenase (*pmoCAB*), the PQQ-dependent methanol dehydrogenases (*mxaFI* and multiple copies of *xoxF*), genes for tetrahydromethanopterin (H_4_MPT)- and tetrahydrofolate (H_4_F)-dependent C_1_-transfer pathways, genes of the ribulose monophosphate pathway, including its phosphoketolase variant (10), and genes encoding a complete tricarboxylic acid (TCA) cycle and a partial serine cycle (10) (Table 1). The *pxmABC* gene clusters (11) linked to a distant homologue of the nitrate-nitrite transporter (*narK*) were found in the *Methylobacter* sp. strain BB5.1 and *M. marinus* A45 genomes. A phosphoenolpyruvate carboxylase gene (*ppc*) was found in *M. vadi* IT-4 only. Genes encoding soluble methane monooxygenase, known glyoxylate regeneration pathways, and RubisCO (*cbbL* and *cbbS*) were not detected. Genes involved in ammonium and nitrate assimilation are present in all three genomes. The genomes of strains A45 and BBA5.1 contain all genes necessary to provide for urea hydrolysis and nitrogen fixation. *M. vadi* IT-4 has the potential for dissimilatory nitrite reduction to nitric oxide, as suggested by the presence of *nir* genes. The NADH:ubiquinone reductase (H^+^)-translocating genes (*nuoABCDEFGHIJKLMN*) were identified in *M. marinus* A45 only. All strains possess genes encoding Na^+^-transporting NADH:ubiquinone oxidoreductase (*nqrABCDEF*), ubiquinol-cytochrome *bc*_1_ complex, cytochrome *b*, cytochrome *c* oxidase, cytochrome P450 and P460, and cytochrome *d* ubiquinol oxidase. Cytochrome *bo*_3_ quinol oxidase was found in *M. vadi* IT-4 only. Both *Methylobacter* species possess genes encoding the Na^+^-translocating ferredoxin:NAD^+^ oxidoreductase complex (*rnfABCDGE*). All genomes contain genes encoding pyruvate-ferredoxin/flavodoxin oxidoreductases, and all three strains possess ectoine biosynthesis genes.

The genome of *M. marinus* A45 includes a chromosomally integrated complete copy of a bacteriophage genome (predicted size, 65 kb) integrated in the chromosome, indicating the possibility of lysogenic infection in methanotrophic bacteria. These genomes provide a valuable resource to obtain new insights into environmental controls of fitness and diversity in methanotrophs, mechanisms of genetic exchange within methanotrophic communities, and the potential for the development of new genetic tools for methanotrophs.

### Nucleotide sequence accession numbers. 

The genome sequences have been deposited in GenBank under the accession numbers listed in Table 1. 
